# Breast Cancer Cells Induce Osteolytic Bone Lesions *In vivo* through a Reduction in Osteoblast Activity in Mice

**DOI:** 10.1371/journal.pone.0068103

**Published:** 2013-09-12

**Authors:** Laura S. Gregory, Wilson Choi, Leslie Burke, Judith A. Clements

**Affiliations:** 1 Institute of Health and Biomedical Innovation, Queensland University of Technology, Brisbane, Queensland, Australia; 2 Skeletal Biology and Forensic Anatomy Research Program, School of Biomedical Sciences, Faculty of Health, Queensland University of Technology, Brisbane, Queensland, Australia; 3 School of Biomedical Sciences, Faculty of Health, Queensland University of Technology, Brisbane, Queensland, Australia; Rutgers - New Jersey Medical School, United States of America

## Abstract

Bone metastases are severely debilitating and have a significant impact on the quality of life of women with metastatic breast cancer. Treatment options are limited and in order to develop more targeted therapies, improved understanding of the complex mechanisms that lead to bone lesion development are warranted. Interestingly, whilst prostate-derived bone metastases are characterised by mixed or osteoblastic lesions, breast-derived bone metastases are characterised by osteolytic lesions, suggesting unique regulatory patterns. This study aimed to measure the changes in bone formation and bone resorption activity at two time-points (18 and 36 days) during development of the bone lesion following intratibial injection of MDA-MB-231 human breast cancer cells into the left tibiae of Severely Combined Immuno-Deficient (SCID) mice. The contralateral tibia was used as a control. Tibiae were extracted and processed for undecalcified histomorphometric analysis. We provide evidence that the early bone loss observed following exposure to MDA-MB-231 cells was due to a significant reduction in mineral apposition rate, rather than increased levels of bone resorption. This suggests that osteoblast activity was impaired in the presence of breast cancer cells, contrary to previous reports of osteoclast-dependent bone loss. Furthermore mRNA expression of Dickkopf Homolog 1 (DKK-1) and Noggin were confirmed in the MDA-MB-231 cell line, both of which antagonise osteoblast regulatory pathways. The observed bone loss following injection of cancer cells was due to an overall thinning of the trabecular bone struts rather than perforation of the bone tissue matrix (as measured by trabecular width and trabecular separation, respectively), suggesting an opportunity to reverse the cancer-induced bone changes. These novel insights into the mechanisms through which osteolytic bone lesions develop may be important in the development of new treatment strategies for metastatic breast cancer patients.

## Introduction

Breast cancer is the most common form of cancer in females worldwide and is the second most common cause of cancer-related deaths for Australian and North American women [1,2]. In approximately 70% of patients in the advanced stages of breast cancer, the cancer cells move preferentially to the skeleton [3]; and once metastasised, survival rate decreases significantly as no cure is currently available [2].

Throughout adult life, the human skeleton is continuously remodelled by microscopic patches of bone resorption (degradation) by osteoclasts, which is coupled with bone formation by osteoblasts at the same site [4]. In healthy bone tissues, these two phases are tightly regulated and occur in a balanced sequence, such that bone tissue quality and bone mass are preserved [5]. The remodelling balance is disrupted when metastatic breast cancer cells invade and grow within the bone microenvironment, resulting in the development of metastatic bone lesions that cause the bones to become fragile and therefore fracture more easily [6]. In most breast cancer patients, the bone lesions which develop are characterised by a decrease in bone mass as a result of tumour growth and are termed osteolytic bone lesions [7]. The lesions that form are debilitating because they are primarily in the load-bearing bones of the body, such as the long bones, vertebral column and bony pelvis [8]. Despite the clinical importance of bone metastasis, the mechanisms that lead to the development of metastatic bone lesions in breast cancer patients are not clearly defined.

Bone histomorphometry is the microscopic analysis of the morphology and organisation of bone tissue. It is commonly used to evaluate metabolic changes in bone tissues including changes in bone density, structural re-organisation of the bone matrix, as well as dynamic measurements of bone formation and bone resorption activities [9,10]. Nevertheless, previous studies in the field of breast cancer bone metastases have failed to utilise histomorphometry effectively to define the changes in bone formation and bone resorption activities that occur during osteolytic bone lesion development with a limited number of studies using endpoint data from post-mortem samples and patients with debilitating fractures [7,11]. Osteoclast number has been analysed repeatedly as a measure of resorptive activity during metastatic lesion development in animal models [12-16] but this form of analysis assumes that all osteoclasts participate equally in the bone resorption process. The proportion of the bone surface undergoing resorption can provide a more accurate method of determining osteoclast activity [9].

Bone loss can occur through three different mechanisms, all of which occur due to unbalancing of the bone remodelling cycle: (i) increase in bone resorption activity; (ii) decrease in bone formation activity; or (iii) a simultaneous increase in bone resorption and a decrease in bone formation. Given that changes to the rate of bone formation have not been subjected to dynamic measurements in previous studies in this field, it is unclear how osteolytic bone lesions develop in breast cancer bone metastases. In most studies, the decrease in bone mass was attributed to an overall increase in bone resorption, as suggested by an increase in osteoclast number [14,15,17,18]. However, recently molecular profiling and osteoblast number analysis has suggested that the bone loss in osteolytic lesions may be due to a concomitant decrease in bone formation activity [14,19,20,21]. Furthermore there is *in vitro* evidence that the co-culturing of MC3T3-E1 pre-osteoblastic cells with MDA-MB-231 breast cancer cells results in a reduction in osteoblast differentiation, adhesion properties, mineralisation and increased apoptosis [22]. Therefore there is compelling evidence that breast cancer cells modulate the function of both osteoblasts and osteoclasts. However in *vivo* studies investigating the effect of breast cancer cells on osteoblast function and bone mineralisation are still lacking.

This study aimed to measure the changes in osteoblastic and osteoclastic activity within bone tissue of immunodeficient mice at two different time-points following intratibial injection of the MDA-MB-231 human breast cancer cell line. From the histomorphometric analysis, there is evidence to suggest that the early stages of osteolytic bone lesion development occurs primarily by retardation of the bone formation response – as indicated by a significant decrease in osteoblastic activity in the cancer cell-injected limb. This contrasts with the current assumption that the decrease in bone mass associated with breast cancer osteolysis is primarily due to increased bone degradation. Although further work is needed with other osteolytic breast cancer cell lines to confirm this observation, the result of this study may have significant implications for the development of treatments that aim to reduce the severity and occurrence of metastatic bone lesions in breast cancer patients.

## Materials and Methods

### Tissue culture

MDA-MB-231 cells (American Type Culture Collection, Rockville, MD, USA) were cultured in DMEM medium (Invitrogen, Mt Waverly, Victoria, Australia) and supplemented with 10% foetal calf serum (Invitrogen) and 100 units/mL penicillin G sodium and 100 µg/ml streptomycin sulphate (Invitrogen). The cells were tested to be free from *Mycoplasma* contamination using the Takara PCR Mycoplasma Detection Set (Takara Bio, Madison, Wisconsin, USA).

MDA-MB-231 cells were grown to 70-80% confluence for viability determination. The cells were detached using EDTA, resuspended and placed on ice to mimic storage of the MDA-MB-231 cells on the day of surgery. The viability of the cells was determined at 30 minute intervals over a 3-hour period using the Trypan Blue exclusion method and a Neubauer haemocytometer (Brand GMBH+ CO KG, Wertheim, Germany).

### Ethics statement and Intratibial injection of cancer cells

Six week old female SCID mice (n=10 per time-point) were housed under a specific pathogen free environment at the Princess Alexandra Hospital Biological Research Facility and had constant access to standard mouse pellets and water. This study received ethical approval from the Queensland University of Technology animal ethics committee (approval number: 070000061) with ratification from the University of Queensland animal ethics committee (#168/07). Mice were provided with enrichment during the course of the study including sunflower foraging and cardboard for shredding and nesting behaviour. All efforts were made to minimise suffering during the surgical procedure.

Prior to surgery, the animals were anaesthetised by intraperitoneal injection of ketamine (Parnell) and xylazil-20 (Ilium) in sterile water (at a ratio of 3:2:25). With the knee joint flexed, a 26-gauge needle was inserted transcutaneously through the patellar ligament and into the proximal tibial metaphysis. Ten microLitres of the cancer cell suspension at a concentration of 5 x 10^6^ cells/mL PBS was injected into the left tibia. PBS alone was injected as a vehicle into the contralateral limb using the same procedure to account for a possible bone adaptation response from tissue irritation caused by the needle entry. Based on the viability assay we found that 80% of the MDA-MB-231 cells were viable 3 hours after detachment from the culture flask, which was sufficient time to complete all surgeries (n=10) each day.

### Radiography

Given that the bone remodelling cycle is around 16-20 days in mice [23], the mice were randomly allocated to two different time-point groups -18 days or 36 days post-injection - to examine changes in bone tissue architecture across two remodelling cycles. For dynamic histomorphometry, the calcium-binding fluorochrome label calcein (1mg/mL; Sigma Chemical Co, St Louis MO, USA) was delivered by intraperitoneal injection at a dose of 10mg/kg into each animal at 7 and 2 days before euthanasia to give a 5 day inter-label period. All mice were euthanized by carbon dioxide overdose and the left and right hindlimb of each animal was imaged using the Kodak Image Station: *in vivo* FX (Eastman Kodak Company, Rochester, NY, USA) to determine whether radiographically visible lesions had formed.

### Resin Embedding and Structural Histomorphometric Analysis

The left and right tibiae were isolated and fixed in 10% neutral buffered formalin and embedded in methyl-methacrylate. Thick longitudinal sections (70-100µm) were cut from each tibia using a diamond-blade microtome (Leitz 1600 Saw Microtome, Leica, Wetzlar) and mounted unstained on glass slides for light and fluorescence microscopy using a Zeiss Axio Imager Z1M fluorescent microscope (Carl Zeiss Inc., North Ryde, NSW, Australia) at a magnification of x200. Digital photomicrographs of the proximal metaphysis were taken using a Zeiss MRc5 camera and AxioVision software (Carl Zeiss Inc.) and analysed using ImageJ software (National Institute of Health, USA). The following variables – abbreviated according to Parfitt et al. [9] – were measured in each section: trabecular area (Tb.Ar); tissue area (T. Ar); trabecular perimeter (Tb.Pm); resorption perimeter (Rs.Pm); double-labelled perimeter (dL.Pm); single-labelled perimeter (sL.Pm); and inter-label width (Ir.L. Wi). Only secondary spongiosa in the proximal tibial metaphysis were measured in a representative region 450µm distal to the epiphyseal growth plate and extending 900µm distally. From this information, a range of histomorphometric indices were calculated using the formulae in Table 1 [24,25].

**Table 1:** Formulae for the calculation of histomorphometric indices and the meaning of each index.

**Histomorphometric index**	**Formula for calculation^A^**		**Interpretation of the index^B^**	
Percentage trabecular area (%Tb.Ar, %)	Tb.Ar/T.Ar x 100	The proportion of tissue area containing trabecular bone tissue.	
Trabecular width (Tb.Wi, μm)	(2000/1.199) x (Tb.Ar/Tb.Pm)	An architectural measurement that describes the average width of each trabecular bone piece.	
Trabecular number (Tb.N, n/mm)	(1.199/2) x (Tb.Pm/T.Ar)	An architectural measurement that describes the number of trabecular bone pieces present in the tissue area. High Tb.N indicates fragmentation of the bone matrix (if Tb.Sp is also high); whereas a low Tb.N indicates connectivity between the trabecular pieces (if Tb.Sp is also low or unchanged).*	
Trabecular separation (Tb.Sp, μm)	(2000/1.199) x (T.Ar – Tb.Ar)/Tb.Pm	An architectural measurement that describes the distance between each trabecular bone piece and indicates the looseness of the bone tissue matrix.*	
Mineralising surface (MS, %)	(dL.Pm + sL.Pm/2)/Tb.Pm x 100	An indicator of the number of active osteoblasts over the bone surface.	
Mineral apposition rate (MAR, μm/day)	Ir.L.Wi/5	An indicator of osteoblast activity by determining the rate at which osteoblasts are laying down new bone matrix.	
Bone formation rate per surface (BFR/BS, μm^2^/μm^3^/d)	MS x MAR x 3.65	An indicator of the overall osteoblast contribution to the observed bone changes and is a combination of both osteoblast number and osteoblast activity.	
Bone formation rate per unit area (BFR/B.Ar, %/y)	(sL.Pm/2 + dL.Pm) x MAR/Tb.Ar x 365 x 100	Considered to be the rate of bone remodelling or bone turnover.	
Resorption surface (Rs.S, %)	Rs.Pm/Tb.Pm x 100	An indicator of osteoclast activity, as determined by the proportion of the bone surface undergoing resorption.	

* Tb.N and Tb. Sp need to be considered together to understand trabecular spatial orientation.

A = Formulae as described by Li et al. 1990 (22)B = Meaning of each index based on Parfitt et al. 1983 (23)

### Paraffin Embedding and Cellular Histological Analysis

An additional three animals were inoculated with MDA-MB-231 cells in the right tibia and sacrificed at 36 days post-injection. Tibiae were extracted, fixed and decalcified in EDTA for paraffin embedding. Thin longitudinal sections (4µm) were cut using a rotary microtome (Leica RM2235) and stained with tartrate resistant acid phosphatase (TRAP) and counterstained with Mayer’s haematoxylin. Osteoclasts were identified as TRAP-positive, multinucleated cells on the trabecular bone surface. The number of osteoclasts per trabecular bone perimeter (N. Oc/Tb.Pm) and percentage of bone surface lined by osteoclasts (Oc.S/BS) was calculated in the proximal metaphysis (same representative region measured as described for structural histomorphometric measurements above) using a Nikon Eclipse Ci Fluorescent Microscope with a x 20 objective and Osteomeasure software (Osteometrics Inc., Atlanta, Georgia, USA). In addition immunostaining with Anti-NuMA primary antibody (rabbit polyclonal anti-human NuMA, 1:100, EPITOMICS®, S2825) and Peroxidase-labelled dextran polymer conjugated goat anti-mouse secondary antibody (Dako, Australia, K406189) was conducted to aid identification of human-specific cells in the mouse, namely the injected MDA-MB-231 cells.

### Statistical Analysis

All histomorphometric data were analysed for statistical significance using the SPSS statistics package (SPSS Inc., Chicago, Illinois) by a one-way analysis of variance where we allocated the factorial treatment combinations to treatments of a single factor with equal variance being assumed. Variance homogeneity was confirmed by determining that the ratio of the largest standard deviation to the smallest standard deviation was not greater than 4 for each dependent variable [26,27]. Significance was accepted at *P* < 0.05, while probability levels between 0.05 and 0.1 were classified as marginal if the difference between the means was greater than twice the standard error of the mean.

### Real time PCR profiling of cell lines

Total RNA was isolated from the MDA-MB-231 cell line (RNA from LNCaP cell line used as a negative control), using TRIzol (Invitrogen) and purified using the RNeasy Mini Kit (QIAGEN, Australia) as per the manufacturers’ instructions. Reverse transcription and cDNA synthesis was performed using Superscript III (Invitrogen). Gene-specific oligonucleotide primers were designed based on cDNA sequences derived from the NCBI sequence database and using Primer Express software (Applied Biosystems, Scoresby, Victoria, Australia). Primers manufactured by Sigma-Proligo (Castle Hill, Australia) were 5’-CCCATCCAGTACCCCATCATT-3’ (sense) and 5’-CCAAGATCCAACTACGAGCTTTTT-3’ (antisense) primer sequences for human noggin and 5’-ACCATTGACAACTACCAGCCGT-3’ (sense) and 5’-GGAATACCCATCCAAGGTGCTAT-3’ (antisense) primer sequences for human DKK-1 expression. PCR reactions were conducted on an ABI Prism 7000 Thermal Cycler (Applied Biosystems) using the SYBR green dye detection system as per the manufacturer’s instructions. For each cell line, the reaction was performed in triplicate for two separate cDNA samples and relative levels of gene expression were normalised to 18S ribosomal RNA primers (5’-GATCCATTGGAGGGCAAGTCT-3’, sense and 5’-CCAAGATCCAACTACGAGCTTTTT-3’, antisense).

## Results

### Qualitative analysis of bone lesion development

**Figure 1:**
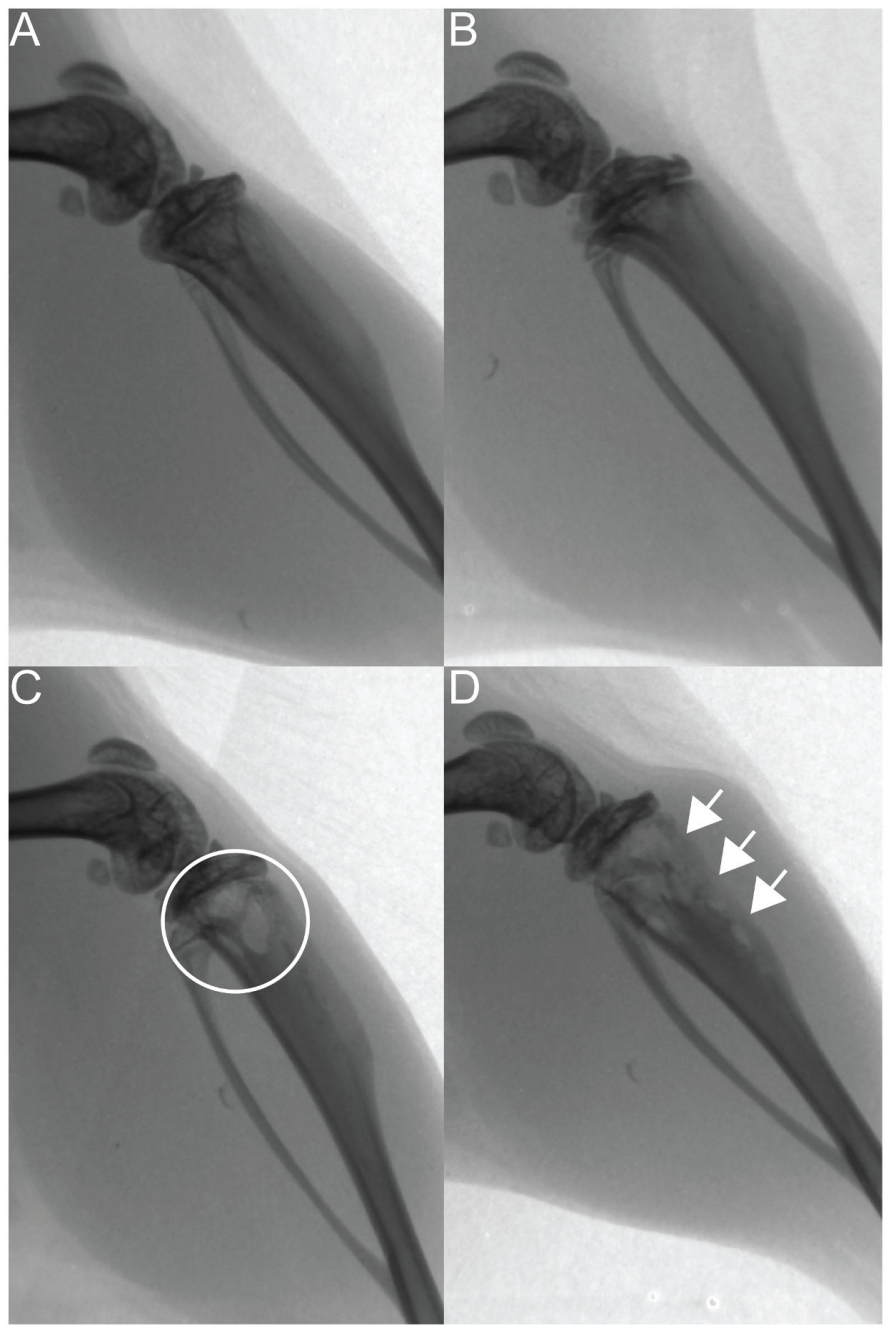
Radiographic appearance of osteolytic tumour lesions resulting from intratibial injection with MDA-MB-231 breast cancer cells. Lateral projection of mouse hindlimb demonstrating establishment of an osteolytic tumour lesion at 18 days (A) and 36 days (B-D) post-injection with PBS (control: B) or MDA-MB-231 cells (C and D). *White* *circle* in C, area with increased bone loss, only seen in 33% of animals at 36 days. *Arrows* in D indicate area of cortical perforation seen in 20% of animals at 36 days.

At sacrifice, an appreciable bone loss was only detected in three animals from the 36 day post-injection group using radiography (Figure 1). Despite this, 15 of the 20 animals had in fact developed osteolytic lesions based on a >20% reduction in the percentage trabecular area (%Tb.Ar) measured by histology. Qualitatively, there was increased blood supply and a reduction in the amount of trabecular bone tissue in the proximal tibial metaphysis of the cancer-injected limb compared to the vehicle control. NuMA immunohistochemical staining demonstrated the presence of multiple small colonies of MDA-MB-231 cells residing in the proximal metaphysis of paraffin embedded samples indicating an early stage of osteolysis at 36 days post-MDA-MB-231 cell injection. In the three animals that developed overt radiographical lesions, the cortical bone of the proximal tibial metaphysis of two mice was completely perforated (Figure 1D), suggesting the invasion of cancer cells into the surrounding soft tissues.

### Quantitative histomorphometric analysis of osteolytic tumour lesions

**Figure 2:**
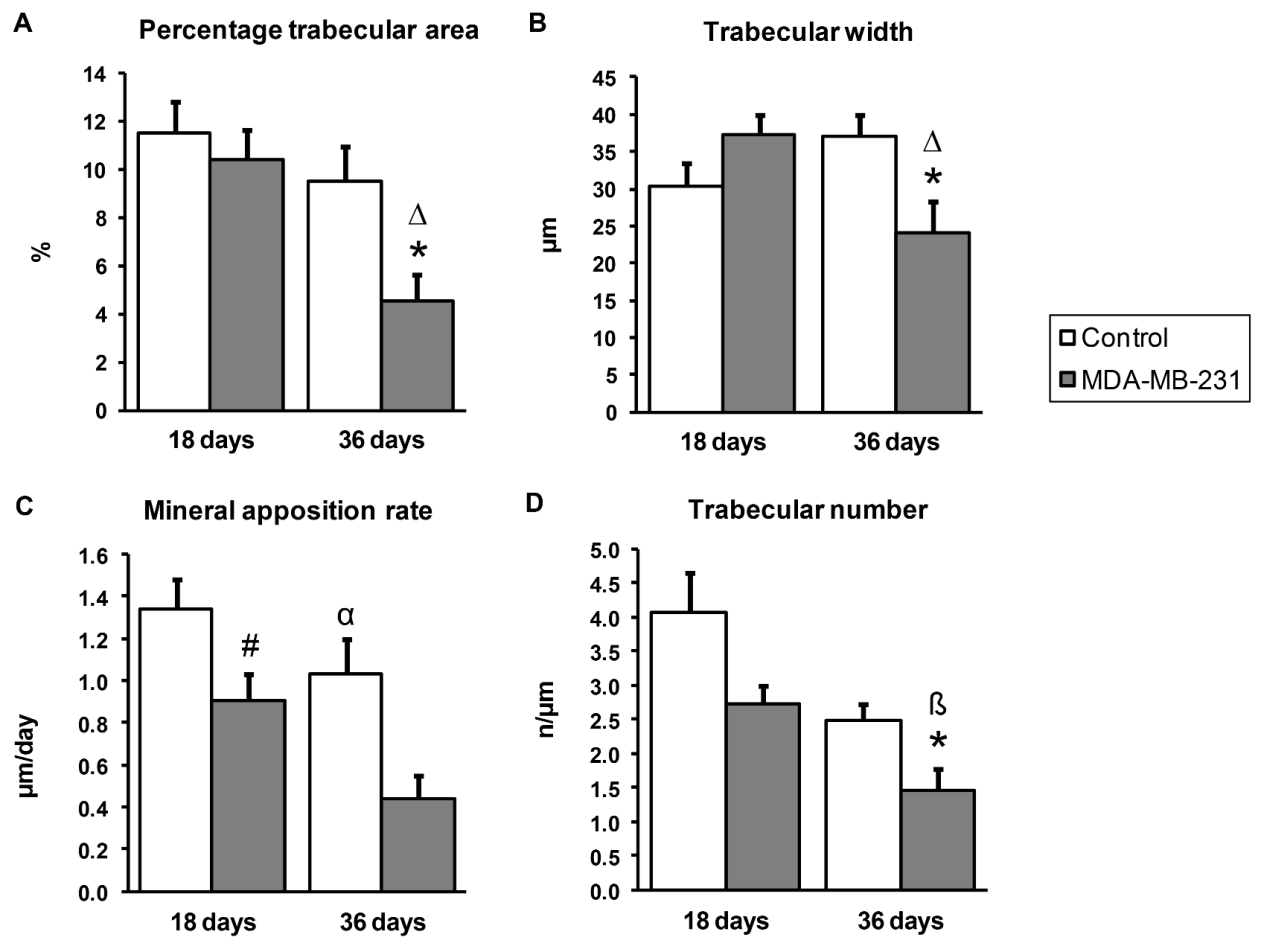
The effect of MDA-MB-231 cells on bone indices at 18 days and 36 days post-surgery. Control limbs were injected with PBS. Values presented are the Mean ± SEM for 10 mice from each time-point. *, significantly different from control limb at 36 days (*p* < 0.05); Δ, significantly different from MDA-MB-231 injected limb at 18 days (*p* < 0.05); ß, marginally different from MDA-MB-231 injected limb at 18 days (*p* < 0.8); #, marginally different from control limb at 18 days (*p* < 0.8); α, significantly different from control limb at 18 days (*p* < 0.05).

Quantitatively, the histomorphometry data (Table 2) indicated that the cancer-injected limb had a statistically significant lower percentage trabecular area (%Tb.Ar) (4.56% ± 1.05) compared to the control limb (9.51% ± 1.45) at 36 days post-injection (*p* = 0.045) and compared to the cancer-injected limb at 18 days post-injection (10.37% ± 1.29; *p* = 0.014). These data indicate that the loss in trabecular bone area within the cancer-injected limbs increased with time and became significant at 36 days post-injection (Figure 2).

**Table 2:** Histomorphometric indices of bone remodelling in the proximal tibial metaphysis of female SCID mice following injection of PBS (Control) or MDA-MB-231 breast cancer cells.

	**Control -18 days**	**MDA-MB-231** **-18 days**	**Control -36 days**	**MDA-MB-231** **-36 days**
**%Tb.Ar (%**)	11.510 ± 1.283	10.367 ± 1.288	9.508 ± 1.454	4.559 ± 1.053 ^*, #^
**Tb. Wi (μm**)	30.334 ± 3.122	37.338 ± 2.583	36.988 ± 2.882	24.026 ± 4.346 ^*, #^
**Tb.N (n/mm**)	4.074 ± 0.579	2.724 ± 0.265^Δ^	2.481 ± 0.256^§^	1.469 ± 0.311
**Tb. Sp (μm**)	255.594 ± 38.570	370.747 ± 51.254	400.885 ± 42.487	488.410 ± 122.229
**MS (%**)	13.209 ± 1.347	14.950 ± 1.090	13.138 ± 2.416	11.956 ± 3.011
**MAR (μm/d**)	1.343 ± 0.134	0.908 ± 0.123	1.035 ± 0.160	0.437 ± 0.111 ^*, ¶^
**%Rs.S (%**)	8.125 ± 1.191	7.790 ± 0.974	6.830 ± 0.906	5.347 ± 1.372
**BFR/BS (μm^2^**/μm**^3^/d**)	63.528 ± 8.566	51.634 ± 8.651	53.573 ± 12.617	28.593 ± 11.231
**BFR/B.Ar (%/y**)	365.044 ± 44.884	237.287 ± 44.058	222.312 ± 46.618	160.278 ± 68.936

The animals were sacrificed at 18 days (time-point 1) or 36 days (time-point 2) post-injection. Values are Mean ± SEM (n = 10). * *p* < 0.05 vs PBS-injected limb at 36 days; #, *p* < 0.05 vs MDA-MB-231 injected limb at 18 days; ¶ *p* = 0.079 vs MDA-MB-231 injected limb at 18 days; Δ, *p* = 0.072 vs PBS-injected limb at 18 days; § *p* < 0.05 vs PBS-injected limb at 18 days.

Associated with this decrease in percentage trabecular area was a significant decrease in the trabecular width (Tb. Wi). The Tb. Wi was significantly lower (*p* = 0.041) in the cancer-injected limb (24.03µm ± 4.35) compared to the control limb (36.99µm ± 2.88) at 36 days post-injection and Tb. Wi significantly declined in the cancer-injected limbs between 18 and 36 days (*p* = 0.035) (Figure 2).

Trabecular number (Tb.N) or fragmentation of the bone matrix was marginally lower in the cancer-injected limbs (2.72n/mm ± 0.27) compared to the control limbs (4.07n/mm ± 0.58) at 18 days post-injection (*p* = 0.072) (Table 2, Figure 2). In addition, the trabecular number had significantly decreased (*p* = 0.025) in the control limbs at 36 days (2.48n/mm ± 0.26) compared to 18 days post-injection. When considering this result in conjunction with no significant change in trabecular separation (Tb. Sp, distance between each trabecular bone piece) being observed, it suggests increased trabecular bridging occurred in the control animals as an age-related phenomenon resulting in increased interconnectivity. The reduction in Tb.N is consistent with the fact that juvenile animals were used in this study and that longitudinal bone growth was still occurring to increase the connectivity between each trabecular bone piece.

No statistical significance was observed in the index mineralising surface (MS) between the cancer-injected and control limbs at both 18 days and 36 days post-injection (Table 2). Given that MS is an indicator of the number of active osteoblasts on the surface of trabecular bone, our observation indicates that the number of osteoblasts participating in bone formation has not changed significantly between the treatment groups.

Interestingly, the mineral apposition rate (MAR) was significantly lower in the cancer-injected limb (0.44µm/d ± 0.11) compared to the control limb (1.04µm/d ± 0.16) at 36 days post-injection (*p* = 0.016) but not at 18 days post-injection (Table 2; Figure 2). There was also a marginally significant decline (*p* = 0.079) in MAR within the cancer-injected limb at 36 days compared with 18 days post-injection (0.91µm/d ± 0.12). This indicates that there was a reduction in the activity of individual osteoblasts in the cancer-injected limb and that this reduction increased with time to become statistically significant at 36 days post-injection. Although there was a trend to suggest that the bone formation rate per surface (BFR/BS) was lower in the cancer-injected limbs compared to the control limbs at 36 days post-injection (Table 2), the difference between the two treatment groups did not reach statistical significance. This is not surprising since this index is calculated by the combined MS and MAR scores and MS was not found to be significantly different between cancer and control limbs.

There was no statistical difference between the cancer-injected and control limbs at both time-points for resorption surface (Table 2), suggesting that osteoclast activity, as indicated by the percentage of trabecular perimeter containing resorption pits, did not differ significantly between the cancer-injected and control limbs. Results from the TRAP-stained bone sections (n=3) resulted in no significant differences in the number of osteoclasts per bone surface or the percentage of bone surface lined by osteoclasts (p>0.18) between the tumour-bearing and control limbs. Interestingly though, there was a trend towards lower osteoclast numbers in the cancer-injected limbs (mean N.Oc/BS 8.90/µm ±2.97 in control limb compared to 4.21/µm ±4.34 in tumour-bearing limb; mean Oc.S/BS 35.34% ±19.98 in control limb compared to 12.26% ±9.56 in tumour-bearing limb) despite the significantly reduced percentage trabecular area seen in these animals; this difference however did not reach significance due to the small sample size allocated to paraffin embedding in this study. Furthermore, no statistical significance was observed in bone formation rate per unit area (BFR/B.Ar) between the cancer-injected and control limbs at both time-points (Table 2), suggesting that the rate of bone remodelling is not significantly affected by the presence of MDA-MB-231 breast cancer cells.

### MDA-MB-231 cells express Noggin and DKK-1 *in vitro*

Given that the observed bone loss in this study was linked to decreased osteoblast activity, and that osteoblast inhibitors have been implicated in pathologic bone loss [28-30], we questioned whether the MDA-MB-231 cells express inhibitors which could potentially suppress osteoblast activity. Specifically, we were interested in the expression of antagonists against the Wnt proteins and bone morphogenetic proteins (BMPs), which are important regulators of osteoblast activity and proliferation [30,31]. With this in mind, we screened the MDA-MB-231 cell line for the Wnt-antagonist, Dickkopf Homolog 1 (DKK-1), and the BMP-antagonist noggin (NOG), using real-time PCR. We also screened the prostate cancer cell line LNCaP as a negative control on the premise that this cell line forms osteoblastic tumour lesions in bone [32]. We found that the MDA-MB-231 cell line expressed DKK-1 and noggin mRNA (1.78x10^-5^ ±1.92x10^-6^ and 4.75x10^-7^ ±7.39x10^-8^ normalised to 18S RNA levels, respectively); whereas the LNCaP cell line did not express these factors.

## Discussion

Breast cancer patients commonly develop metastatic bone lesions which are severely debilitating.Despite its clinical significance, the mechanisms through which breast cancer cells interact with the bone microenvironment to drive the development of bone loss are only starting to emerge in the literature. Previous studies have failed to clarify the changes to the rates of bone formation and bone degradation in the presence of breast cancer cells. Using bone histomorphometry, we report for the first time the temporal changes to the activities of the bone-forming osteoblasts and the bone-resorbing osteoclasts during the development of osteolytic tumour lesions in an animal model using intratibial injection of MDA-MB-231 cells.

In our model, 15 of the 20 animals had an appreciable decrease in %Tb.Ar in the cancer-injected limbs compared to their respective controls as determined by histomorphometry. Nevertheless, most animals developed lesions that were difficult to observe from radiography with only three animals developing radiographically overt lesions, in which a significant degree of trabecular bone loss and cortical bone perforation could be observed macroscopically. This suggests that plain radiography is not sufficiently sensitive to detect early changes in trabecular bone mass. Nevertheless, in most animal models of breast cancer bone metastasis, the degree of bone loss has been determined by measuring the osteolytic lesion area on radiographs using computer-automated grey-level density measurements [15,16,33]. This is an important limitation to their estimated values, because to obtain accurate and quantitative measures of trabecular bone loss, particularly for subtle changes, bone histomorphometry is required.

Results from the histomorphometric analysis indicated that there was a significant reduction in trabecular bone area in the presence of MDA-MB-231 cells at 36 days post-injection and that the extent of bone loss increased with time; which supports the findings of previous studies [13,34] where a decrease in trabecular bone area was observed following intratibial implantation of MDA-MB-231 cells. Our results also indicate that significant evidence of bone loss only occurred following two bone remodelling cycles, given that there was no significant reduction in %Tb.Ar in the cancer-injected limb at 18 days post-injection. Previous studies have demonstrated that angiogenesis is a necessary process for the growth of MDA-MB-231 tumour masses in bone and the subsequent osteolysis [17,35,36]. Interestingly, we observed increased vascularisation in the cancer-injected limbs at 18 days post-injection, suggesting that MDA-MB-231 cells were in the early stages of tumour formation where blood vessels are important for continual growth.

In our model, the observed bone loss was linked to a marked reduction in osteoblast activity. Even though the number of active osteoblasts did not differ significantly (as indicated by no change in mineralising surface), the rate of bone mineral deposition of individual osteoblasts (as indicated by mineral apposition rate) was significantly lower in the cancer-injected limbs at 36 days post-injection compared to their respective controls. In addition, our data has shown that osteoclast activity (as indicated by percentage resorption surface) and osteoclast number (as indicated by osteoclast number per bone surface) did not differ significantly between the cancer-injected and PBS-injected limbs. In fact a trend was observed in our data where osteoclast number and activity is reduced in the presence of breast cancer cells. Our results are supported by Phadke et al. [14] who report a significant decrease in both osteoclast and osteoblast numbers at 4 weeks post-MDA-MB-435 cell injection. Taken together, these results suggest that the observed bone loss in our study was due to decreased osteoblast activity, rather than increased osteoclast activity.

Our finding is in contrast with experimental findings which suggest that the increased bone loss was due to increased osteoclastic activity. A number of *in vitro* studies have shown that breast cancer cells secrete a range of factors that contribute to osteoclast differentiation and proliferation [12,37-39]. However an increased number of osteoclasts [12-16] does not necessarily result in an increase in resorption activity. Moreover, one cannot conclude that the bone loss is osteoclast-dependent without measurements of the osteoblast activity, given that a decrease in bone formation activity can contribute to a reduction in trabecular bone area [25]. Therefore our study, through examination of osteoblast and osteoclast activity, highlights that it is through a significant downregulation of osteoblast activity that bone loss in the breast cancer-induced bone lesion *in vivo* is induced.

Brown et al. [21] demonstrate that the behaviour of both osteoblasts and osteoclasts is dependent on the proximity and size of the breast cancer tumour mass within the bone microenvironment. Therefore the activity of bone cells changes with advancing osteolytic progression. This makes cross-study comparison challenging when results are reported across a small number of timepoints. The observation of only two mice exhibiting severe cortical perforations in our study indicates that the majority of mice were in the early stages of osteolytic development. As the focus of this study was to measure the dynamic histomorphometric changes of the osteolysis development, undecalcified sections were required, limiting the visibility of adjacent tumour tissue. NuMA (nuclear mitotic apparatus protein) immunohistochemical staining was performed in the small sample of paraffin-embedded bones. It confirmed the presence of small colonies of breast-cancer cells in the proximal metaphysis of the tibia, supporting that the majority of mice in our study were in the early stages of osteolytic disease. Therefore our finding that trabecular bone loss has developed through a significant retardation of osteoblast activity, is specific to the early stages of osteolysis. Cortical perforations associated with late stage osteolysis and large tumour colonies that often fill the entire medullary cavity are likely driven by modulation of osteoclast behaviour [21] but may occur through resorption by the tumour cells themselves [14].

The rate of bone turnover did not differ significantly between the cancer-injected and control limbs at both time-points, indicating that the observed bone loss could not be a temporary change associated with increased bone turnover. In physiological bone remodelling, the removal of old bone matrix and subsequent filling of resorption pits with new bone can take approximately 16-20 days in mice [23] and during this remodelling period the bone tissue can appear to be undergoing bone loss because the resorption pits are yet to be filled. However in our model, the reduction in trabecular area was due to uncoupling of normal bone remodelling evidenced by the decrease in activity of osteoblasts and unchanged level of bone resorption, which led to a relatively higher level of bone degradation activity and the development of an osteolytic lesion.

Results from the histomorphometric analysis have shown that the bone loss observed was due to an overall thinning of the trabecular bone pieces rather than perforation of the bone tissue matrix. This is supported by our finding that the osteoclast activity did not differ significantly in the presence of breast cancer cells. An increase in resorption activity is likely to perforate the trabecular struts and would have led to increased trabecular separation [25,40], which was not the case in our study. In contrast, if each resorption pit was not completely filled due to insufficient osteoblast activity, then there would be a gradual thinning of the trabecular bone pieces with time following each remodelling cycle. Indeed, this is what we have observed.

Bone loss becomes irreversible when the trabecular pieces are lost, because there is no platform for the bone-forming osteoblasts to work on to repair the damaged tissue [25]. New pieces of trabecular bone can only be formed during endochondral ossification during the juvenile growth period. After this point perforations in the bone tissue matrix are difficult to repair and structural integrity can be permanently compromised. In this regard, our results have significant implications because it appears that the bone loss associated with MDA-MB-231 cell infiltration was due to overall thinning of each trabecular bone piece, rather than initial perforation of the trabecular architecture which would have otherwise destroyed the interconnections between the trabecular bone pieces. This suggests that, at least in the early stages of the development of breast cancer bone metastasis where the connectivity in bone tissue matrix is preserved, the bone loss is theoretically reversible.

Currently, much research in breast cancer metastasis focuses on targeting the osteoclast population and/or bone resorption with the success of therapeutic targets being gauged by their capacity to control the level of osteoclast activity [41,42]. Bisphosphonates are the current gold standard of care for breast cancer patients with bone metastases [43]. They are a class of anti-catabolic drugs which suppress bone resorption activity by inducing osteoclast apoptosis or by inhibiting osteoclast function [44]. Large scale clinical trials have demonstrated that bisphosphonates reduce tumour burden and prevent further bone loss in animal models [16,18] as well as reducing bone pain in patients with breast cancer metastasis [45] by targeting the tumour cells, angiogenesis and the bone microenvironment. However the effectiveness of bisphosphonate treatment in bone metastasis has been demonstrated to be lower than that seen in osteoporosis therapy, as soluble factors released from breast cancer cells can interfere with the action of the bisphosphonate in inducing osteoclast apoptosis [46]. Furthermore bisphosphonate treatments merely maintain bone mass without restoring the bone that has been lost [48] and patients continue to develop pathologic fractures. This may be because there is insufficient bone formation activity at the metastatic site to regenerate the bone which has been lost, as suggested by our findings. Early clinical trials with denosumab, a human monoclonal antibody that neutralises receptor activator of NF-kappa-B ligand (RANKL), have shown promising results with reduced bone resorption, increased bone mineral density and reduced risk of fracture in osteoporosis and bone metastasis patients [47]. However despite its FDA approval in 2010, there are ongoing concerns regarding a high incidence of hospitalisations due to infections in denosumab trials [48]. RANKL binding is not only required for osteoclast formation but also immunogenesis and therefore denosumab may be producing adverse effects in the differentiation of T and B cells [49]. Much work is therefore still needed to identify safe and effective treatments for bone metastasis that target both the bone microenvironment and the tumour cells directly. In this regard, the administration of anabolic drugs which stimulate the osteoblasts to increase rates of new bone formation is one direction that has not been explored in the treatment of breast cancer bone metastases, given their potential to stimulate the re-building of bone that has been lost.

We have demonstrated using real time PCR that the MDA-MB-231 cell line expresses the mRNA for noggin and DKK-1. This is consistent with recent studies which have demonstrated that DKK-1 and noggin are expressed in high levels in human clinical samples of breast cancer bone metastases [19,20,48,50], with DKK-1 expression being significantly lower in osteoblastic metastatic lesions and in breast cancer patients with non-bone metastases [20]. Previously, the Wnt-antagonist DKK-1 has been implicated in reduced bone formation activity in multiple myeloma [28] and in animal models of prostate cancer bone metastasis [29] where it may regulate the transition between osteolytic and osteoblastic phenotypes [51]. In fact some studies have indicated that Wnt signalling is not only important for osteoblastic differentiation but also can inhibit osteoprotegerin secretion by osteoblasts and therefore may lead to increased osteoclastic activity [20,50]. In our study however, we did not observe increased osteoclastic activity from our histomorphometric analysis. In addition, the BMP-antagonist noggin appears to facilitate decreased bone formation in an animal model of prostate cancer bone metastasis (30) and in a number of osteolytic cell lines *in vitro* with prostate-derived cancer cell lines expressing higher levels of noggin than breast cancer derived cells [52]. Schwaninger et al. [52] confirm in their study that through molecular expression analysis it appears that both inhibition of osteoblast activity and stimulation of osteoclast recruitment are necessary for the full expression of the osteolytic metastatic phenotype. Interestingly Brown et al. [21] have demonstrated that whilst only the osteoclastic cell populations adjacent to breast cancer cells were significantly increased, only the osteoblastic cell populations not in contact with breast cancer cells were significantly modulated. This suggests contrasting signalling patterns between bone cell type and breast cancer cells. Furthermore Bu et al. [50] have demonstrated that the addition of conditioned media from MDA-MB-231 cells inhibits Wnt3A-induced osteoblast differentiation of C2C12 cells. Therefore the tumour-induced osteoblast regulation appears to rely on secreted factors from the cancer cells, further supporting the important role of paracrine molecules such as DKK-1 and Noggin in modulation of osteoblast function by breast cancer cells. Therefore our results complement these *in vitro* studies, where we show for the first time significant down-regulation of osteoblast activity in the bone remodelling cycle in the presence of MDA-MB-231 cells *in vivo*.

It is important to note that the MDA-MB-231 cell line represents only a subpopulation of breast cancer cells that have the potential to induce osteolytic bone lesions. Given that only one cell line was used in this study, it would be premature to conclude whether our findings using MDA-MB-231 cells represent the general mechanism through which all osteolytic bone lesions develop in breast cancer metastases. However MDA-MB-231 cells are currently the only commercially available breast cancer cell line that has been shown to induce purely osteolytic bone changes and have a high metastatic rate to bone [42,53-56]. In fact to date, there are no breast cancer cell lines available that are derived directly from a human bone metastasis [57].

In summary, this study has demonstrated that in our intratibial injection model of MDA-MB-231 cells in SCID mice, the osteolytic bone lesion developed through a decrease in mineral apposition rate and therefore reduced osteoblastic activity, rather than increased bone resorption as commonly reported in the literature. This reduction in osteoblast activity is consistent with the expression of two specific osteoblast inhibitors, DKK-1 and noggin, in MDA-MB-231 cells. In addition, we have determined that the cancer-induced bone loss was due to trabecular thinning rather than perforations of the bone tissue matrix in the early stages of lesion development, which suggests that this bone loss may be reversible. Whilst the osteoblast as a potential therapeutic target is acknowledged in osteoblastic lesions that are common in prostate cancer, there is little mention of the importance of osteoblasts in the development of osteolytic lesions in breast cancer. Thus, our data have provided novel insights into the mechanisms through which breast cancer cells induce osteolytic lesions in bone and may aid in the ongoing development of appropriate treatments.
